# Willingness to Use and Adhere to HIV Pre-Exposure Prophylaxis (PrEP) among Men Who Have Sex with Men (MSM) in China

**DOI:** 10.3390/ijerph16142620

**Published:** 2019-07-23

**Authors:** Liping Peng, Wangnan Cao, Jing Gu, Chun Hao, Jibin Li, Dannuo Wei, Jinghua Li

**Affiliations:** 1 School of Public Health, Sun Yat-sen University, No.74, Zhongshan second road, Guangzhou 510080, China; 2 Department of Health Services, Policy and Practice, School of Public Health, Brown University, Providence, RI 02912, USA; 3 Sun Yat-sen Global Health Institute, Sun Yat-sen University, Guangzhou 510080, China; 4 Department of Clinical Research, Sun Yat-sen University Cancer Center, Guangzhou 510060, China; 5 State Key Laboratory of Oncology in South China, Sun Yat-sen University Cancer Center, Guangzhou 510060, China; 6 Collaborative Innovation Center for Cancer Medicine, Sun Yat-sen University Cancer Center, Guangzhou 510060, China

**Keywords:** pre-exposure prophylaxis, men who have sex with men, HIV prevention, willingness, adherence, China

## Abstract

This study aimed to investigate the levels of willingness to use pre-exposure prophylaxis (PrEP) and intention to adhere to PrEP and to further explore factors associated with PrEP uptake among men who have sex with men (MSM) in China. A total of 524 MSM were recruited from Chengdu, China. Half of the participants had heard of PrEP, and the awareness rate varied from 33.8%, 30.7%, and 7.1% for daily oral PrEP, on-demand PrEP, and long-acting injectable PrEP (LAI-PrEP), respectively. The overall willingness to use any type of PrEP in the next six months was 84.9% if PrEP is effective and provided for free. Participants were less likely to say that they would use PrEP if they used a condom consistently with their regular partners. However, participants were more likely to say that they would use PrEP if they had casual partners in the past month and had higher HIV prevention literacy. The majority of participants intended to adhere to PrEP prescription. More than forty percent (43.1%) of the participants reported that they might reduce condom use if they took PrEP. We found that the overall willingness to use PrEP was high among MSM living in China, but willingness varied across the different types of PrEP.

## 1. Introduction

Men who have sex with men (MSM) are considered to be a high-risk population for HIV infection. Over a quarter of new cases of HIV infection were attributed to homosexual transmission in China [1]. The HIV prevalence among MSM in China increased from 5.5% in 2009 to 7.75% in 2017 [2], and the estimated HIV incidence was over 5 per 100 person-years [3,4]. Despite the promotion of condom use and provision of free HIV testing and antiretroviral therapy (ART) services, the HIV epidemic among MSM has not yet been fully controlled.

Pre-exposure prophylaxis (PrEP) is regarded as a promising preventive strategy for HIV infection, and its regimen is a single pill composed of a compound of antiretroviral drugs (tenofovir disoproxil fumarate with emtricitabine, TDF/FTC). On-demand PrEP refers to taking two pills of oral TDF/FTC 2–24 h before intercourse and then taking one pill of TDF/FTC 24–48 h after intercourse. A randomized placebo-controlled study (IPERGAY) demonstrated that on-demand PrEP can reduce 86% of new HIV infections [5]. No safety difference was found between daily oral PrEP and on-demand PrEP [6]. Long-acting injectable PrEP (LAI-PrEP) refers to a new method of taking PrEP for HIV prevention by injecting drugs with a longer half-life, which can reduce medication frequency (injection every 4–8 weeks). Long-acting cabotegravir (CAB) and long-acting rilpivirine (RPV) are advanced in research. Cabotegravir is an investigational HIV integrase inhibitor, and rilpivirine is a non-nucleoside reverse transcriptase inhibitor; both are crystalline, water-insoluble nanoformulations for intramuscular injection. Long-acting CAB is currently being tested in phase 3 clinical trials (HPTN study 083, NCT02720094) to compare the HIV prevention efficacy of daily oral PrEP with long-acting CAB injected every eight weeks among MSM and transgender women [7].

Given the demonstrated safety and efficacy of PrEP [8], since 2012, the World Health Organization (WHO) has recommended PrEP for individuals at high risk of HIV infection, including MSM [9]. Nearly 40 countries have followed this recommendation and incorporated PrEP into their health and service systems. Among them, 10 countries have implemented nation-wide programs, and the other 30 countries are conducting small-scale projects that are mainly exploring service delivery options, cost, and acceptability of PrEP in preparation for a rollout [10]. In China, PrEP has not been integrated into a nation-wide program [11].

Despite the low awareness (11–33%) of PrEP among Chinese MSM [12,13,14], the willingness to use PrEP was found to be high (64–92%) after briefing on the safety and efficacy of PrEP [15,16]. Most of these acceptability studies investigated daily oral PrEP. Similar studies on other types of PrEP, including on-demand PrEP and LAI-PrEP, are lacking. To prevent HIV infection, on-demand PrEP refers to taking ARV before and after sex, while LAI-PrEP refers to injecting long-acting drugs every 4–12 weeks. The willingness to use PrEP may vary across methods (oral vs. injecting) and frequencies (daily vs. monthly), but factors associated with this willingness have rarely been investigated, particularly among Chinese MSM.

The effectiveness of PrEP is highly dependent on PrEP adherence, which ranges from 44% to 97% in real settings [8,17,18] globally. However, previous studies on adherence to PrEP usage mostly focused on daily oral PrEP. To our knowledge, only one study has been conducted in China on this subject, and it reported that nearly 30% of the participants taking daily oral PrEP had adherence lower than 40% [19]. A multicenter, real-world study on two oral Truvada approaches (i.e., daily oral PrEP and event-driven PrEP) to prevent HIV infection among MSM in China is still ongoing until the end of 2020. Therefore, it is more feasible to assess the intention to adhere to PrEP if they were to take PrEP, which is a strong indicator of actual behavior according to the Theory of Planned Behavior (TPB) [20,21].

Therefore, the aim of the present study was to investigate the level of willingness to use three types of PrEP—daily oral PrEP, on-demand PrEP, and LAI-PrEP—among Chinese MSM and their associated factors. This study also investigated the level of intention to adhere to PrEP usage with these three types and the associated factors. Findings on the willingness to use and intention to adhere to different types of PrEP could provide direct evidence for policy-making and service planning in terms of PrEP implementation among MSM in China.

## 2. Materials and Methods

### 2.1. Participants and Procedure

Participants were recruited from a local gay-friendly health consulting service center in Chengdu between November 2018 and March 2019. Participants were service users of the center. The inclusion criteria were as follows: (1) male aged 18 and above, (2) self-reported anal intercourse with at least one man in the last six months, and (3) self-reported HIV-negative or serostatus unknown. Two experienced peers from the center were hired to facilitate the participants’ recruitment and data collection. Participants were briefed about the study’s purpose and then provided written informed consent before completing a self-administered survey questionnaire. Fieldworkers checked the questionnaire upon completion and offered each participant CNY 50 (about USD 6) as a compensation for their time spent on the survey. The questionnaire took an average of 20 min to complete. A total of 650 eligible participants were approached, of whom 524 (response rate of 80.6%) completed the survey. Ethics approval was obtained from the ethics committee of Sun Yat-sen University ((2018) 049).

### 2.2. Measurement

#### 2.2.1. Background

Sociodemographic information collected included age, ethnicity, marital status, education level, personal monthly income, job type, sexual orientation, and history of sexually transmitted diseases (STDs). Participants were asked to rate their perceived risk of HIV and STD infection on a five-point Likert scale from 1 (very high) to 5 (very low).

Alcohol consumption was assessed by the frequency of drinking in the past year; no alcohol consumption or only 1–2 drinks on special occasions in the past year were classified as non-drinking, and the remaining responses were classified as drinking. Participants were also asked to report their status of binge drinking, which was defined as drinking more than 750 mL of red wine, or 1800 mL of beer, or 150 milliliters of spirits within 2 h more than one day per week in the past month.

#### 2.2.2. Risky Sexual Behaviors

Participants’ sexual behaviors were measured, including the age at which they began having sex with men, number of regular and non-regular sexual partners in the past six months, condom use with regular and non-regular partners in the past month, and roles in sexual intercourse (i.e., insertive, receptive, and both).

#### 2.2.3. HIV Literacy

A 12-item scale developed for this study was used to assess HIV literacy among participants. It consisted of three subscales: literacy related to HIV prevention (3 items), diagnosis (3 items), and treatment (6 items). The scale has the advantage of including the most up-to-date information about HIV, such as “people living with HIV cannot transmit HIV to a partner if their viral load had reached undetectable levels” and “immediate ART initiation is recommended once diagnosed”. Participants were asked to assess whether each statement was correct or incorrect. An example item for HIV prevention subscale was “condoms are effective in preventing the spread of HIV”. An example item for HIV diagnosis subscale was “people infected with HIV will become symptomatic soon”. An example for HIV treatment subscale was “people living with HIV on effective treatment (i.e., having undetectable viral load) cannot transmit HIV to a partner”. HIV literacy was a summary score calculated by adding up each correct answers, with a higher score indicating a higher level of HIV literacy.

#### 2.2.4. PrEP-Related Variables

Awareness of PrEP: Participants were asked whether they had heard of PrEP before the survey. Those who had heard of PrEP were further asked about which types of PrEP they had heard of. Participants were provided with the names, the method (oral or injection), and the frequency of each category (i.e., daily oral PrEP, on-demand oral PrEP, and LAI-PrEP) and were asked to respond “yes” or “no” if they had heard of each.

Conditional willingness to use PrEP: A research assistant gave a 3-min introduction on the three types of PrEP to the participants, including names of the ARV involved, the method (oral or injection) and frequency (daily or non-daily) of PrEP use, and the potential benefits/risks of using these PrEP. For LAI-PrEP, the injections were presented as occurring at 8-week intervals. Participants were asked about their willingness to start using PrEP in the next six months on the condition that “PrEP can reduce the risk of HIV infection by up to 90%” and that “PrEP can be provided for free”. The willingness to use the abovementioned three types of PrEP was asked respectively on a 5-point Likert scale (1 = definitely will not, 2 = probably will not, 3 = uncertain, 4 = probably will, 5 = definitely will). Participants were classified into the “unwilling to use PrEP” group when they endorsed responses 1, 2, or 3 and into the “willing to use PrEP” group when they endorsed responses 4 or 5. The overall willingness to use PrEP was defined as any willingness to use PrEP with one or more of the three types.

Intention to adhere to PrEP: Intention to adhere to PrEP was measured by a single item. Participants were asked, “If you were to take PrEP, would you stick to it as strictly as you were prescribed?” Response options were a 5-point Likert scale (1 = definitely not, 2 = probably not, 3 = uncertain, 4 = probably will, 5 = definitely will). Choices 1, 2, and 3 were designated “low level of intention to adhere” and choices 4 and 5 were designated “high level of intention to adhere”. Self-reported intention to adhere to PrEP was asked for each of the three types of PrEP usage.

Risk compensation following PrEP usage: Participants were asked, “Are you likely to reduce condom use during anal sex after taking PrEP?” Responses were definitely not, probably not, uncertain, probably will, and definitely will. Risk compensation was categorized as “yes” when they responded with uncertain, probably will, or definitely will and as “no” when they responded with probably not or definitely not.

Acceptable price for PrEP: Acceptable price for PrEP was collected using the following single item: “If PrEP is not free, what is the maximum amount you are willing to pay per month (CNY)?” Responses were <100, 100–200, 201–400, 401–600, 601–800, 801–1000, 1001–2000, and >2000.

### 2.3. Statistical Analysis

Willingness to use PrEP and intention to adhere to PrEP were analyzed as two dependent variables, while odds ratios of background variables and other variables (i.e., risky sexual behaviors and HIV literacy) were first presented in the univariate analysis (ORu). Then, we assessed associations between independent variables and two dependent variables while adjusting for potential sociodemographic confounders in the multivariate logistic regression analysis, and the adjusted odds ratios (AOR) and their 95% confidence interval (95% CI) were obtained. All statistical analyses were performed using IBM SPSS Statistics (version 21, SPSS Inc., Chicago, IL, USA), and two-tailed *p* < 0.05 was considered as statistically significant.

## 3. Result

### 3.1. Background Characteristics

Around half (50.9%) of the participants were younger than 25 years old. The mean age was 27.65 (± 8.1). The majority of participants were of Han ethnicity (96.6%), were unmarried (91.0%), had sexual orientation as homosexual (77.11%), had obtained bachelor degree or above (61.5%), and had full-time jobs (64.5%). The majority (94.3%) of participants was HIV-negative, and 5.7% of the participants did not know their own HIV status. Approximately ten percent (7.4%) of the participants reported a history of STDs. Less than one-fifth (17.6%) of the participants perceived a high/very high risk of HIV infection, while 14.1% of the participants perceived a high/very high risk of STDs. Regarding alcohol consumption, about half of the participants (46.9%) were drinking, and 18.5% were binge drinking (Table 1).

### 3.2. Risky Sexual Behavior Characteristics

The mean age of first homosexual intercourse was 21.58 years old (±6.10). Most of the participants reported having 1–2 sexual partners in the past six months. About 76.0% of the participants reported having regular partners in the past six months, and 40.2% of them had regular partners only. More than sixty percent (67.9%) of the participants reported having non-regular partners in the past six months; 66.9% of them also had regular partners. The majority of participants reported consistent condom use during anal intercourse with both regular partners (62.0%) and non-regular partners (75.4%) in the past month (Table 1).

### 3.3. HIV Literacy

The mean score of the HIV literacy scale was 9.4 (SD = 1.89), ranging from 0 to 12. The majority (84.2%) of participants scored higher than 7 (Table 1).

### 3.4. PrEP-Related Characteristics

Half of the participants had heard of PrEP before this study, and the awareness rate was 33.8%, 30.7%, and 7.1% for daily oral PrEP, on-demand PrEP, and LAI-PrEP, respectively. The overall willingness to use any type of PrEP in the next six months was 84.9%, with the willingness rate of 60.1% for daily oral PrEP, 79.2% for on-demand PrEP, and 62.8% for LAI-PrEP. The majority of participants intended to adhere to PrEP if taking PrEP, with the intention rate of 70.2% for daily oral PrEP, 84.9% for on-demand PrEP, and 71.2% for LAI-PrEP. Over forty percent (43.1%) of the participants reported that they might reduce condom use if they start taking PrEP. If PrEP was not free, 60.1% of the participants would be willing to pay CNY100–600 per month (Table 2).

### 3.5. Factors Associated with the Willingness to Use PrEP

In the univariate analysis, younger age, homosexual identity, and drinking were significantly associated with the willingness to use PrEP (univariate odds ratio (ORu) are shown in Table 3). Other background variables, such as ethnicity, education level, marital status, personal monthly income, and job type were not significantly associated with the willingness to use PrEP among this sample.

Adjusted for two background variables (sexual orientation and age), two variables were significantly and positively associated with the willingness to use PrEP: having more than five non-regular partners in the past six months (AOR = 3.36; 95% CI: 1.10–10.26) and possessing a higher literacy in HIV prevention (AOR = 1.49; 95% CI: 1.03–2.16). Consistent condom use during sexual intercourse with regular partners (AOR = 0.47; 95% CI: 0.23–0.95) was significantly and negatively associated with the willingness to use PrEP. Drinking (AOR = 1.56; 95% CI: 0.95–2.56) and having heard of PrEP (AOR = 1.63; 95% CI: 0.99–2.68) were marginally and positively associated with the willingness to use PrEP, while having first homosexual intercourse at an older age (AOR = 0.47; 95% CI: 0.21–1.07, reference group: age under 18 years old) was marginally and negatively associated with willingness. The rest of the tested variables were not significantly associated with the willingness to use PrEP among this sample.

### 3.6. Factors Associated with the Intention to Adhere to PrEP

Adjusted for age and personal monthly income, having heard of PrEP (AOR = 1.67; 95% CI: 1.14–2.44) was positively associated with intended high adherence to daily oral PrEP. Consistent condom use during sexual intercourse with regular partners (AOR = 0.48; 95% CI: 0.24–0.98) was negatively associated with intended high adherence to on-demand PrEP. Possessing higher overall HIV literacy (AOR = 1.13; 95% CI: 1.00–1.26) was positively associated with intended high adherence to on-demand PrEP. Self-reported less likely to reduce condom use after taking PrEP (less risk compensation) was marginally associated with both intended high adherence to daily oral PrEP and LAI-PrEP (Table 4 and Table 5).

## 4. Discussion

This is one of the first studies to investigate the willingness to use three types of PrEP and the intention to adhere to PrEP usage among Chinese MSM. We found the overall willingness to use free PrEP to be high but varied across the different types of PrEP. Number of non-regular partners, condom use with regular partners, and possessing a higher HIV literacy were associated with willingness to use PrEP, while condom use with regular partners, higher HIV literacy and PrEP awareness were associated with intention to adhere to PrEP. The overall awareness rate of PrEP in the present sample was 52.7%, with higher awareness for daily oral PrEP and on-demand PrEP. These awareness estimates are higher than results reported by similar studies in mainland China, which ranged from 11.20% to 22.40% [12,22,23]. This may be because participants in our study were recruited from a gay-friendly health consulting service center. People who take the initiative to seek health services in the center may have a higher level of knowledge and are more likely to have access to information about new biomedical intervention for HIV.

The overall willingness to use PrEP among this Chinese MSM sample was over 80%, which was higher than the estimation (57.8% [24]) of PrEP willingness among global MSM. Higher willingness may be due to the fact we used a setting that PrEP can be provided for free and can reduce the risk of HIV infection by up to 90%. Among the participants who were willing to use PrEP in our study, 8.5% of them were unwilling to self-pay for PrEP and 82.0% were willing to self-pay more than CNY100 per month. We found that having sex with more non-regular partners and inconsistent condom use with regular partners were associated with a higher willingness to use PrEP, which are consistent with previous studies [25,26]. MSM presenting higher risky sexual behaviors were more willing to use PrEP. Despite the high willingness rate, PrEP uptake that results from this willingness might be low, which has been evidenced by previous implementation PrEP studies [27,28,29]. A study conducted among MSM living in Shanghai, China, reported that the actual PrEP uptake rate was only 2.5% when the willingness rate to take them was 19% [30]. Potential reasons to explain this gap include concerns about long-term side effect of ARV, difficulty adhering to medication, and the financial cost of PrEP. Although the safety of PrEP has been demonstrated, PrEP still has side effects, such as gastrointestinal intolerance and renal toxicity. In the real setting, PrEP is not provided for free and is around USD 670 overseas and CNY 2000 per month in mainland China, which is a large financial burden for MSM to access PrEP [31,32,33]. However, with the expiration of drug (TDF) patents, the increase in market competition, and the decrease in price of TDF in China (from around CNY 1500 to 500 per month) [34], a substantial decline in the price of PrEP is expected in the future in China. Recently, the motivational PrEP cascade was put forward and used as a conceptual tool for identifying discrepancies between willingness and behavior [35,36]. The motivational PrEP cascade involves five stages: (1) Stage 1, unwilling to take PrEP, (2) Stage 2, willing to take PrEP, (3) Stage 3, in PrEParation (i.e., seeing PrEP as accessible and planning to initiate PrEP), (4) Stage 4, in PrEP action (i.e., prescribed PrEP), and (5) Stage 5, maintenance and adherence. In the PrEP cascade, willingness to use PrEP is the second stage and is prior to the stages of intention to use PrEP and PrEP uptake. Therefore, even though a significant intention–behavior gap may exist, the finding from the present study can still make a contribution to the field and for service planning. Future studies are needed to explore what the facilitators and barriers are for translating willingness to actual PrEP uptake behavior.

Participants’ willingness to use PrEP varied across the different types of PrEP. Daily PrEP raised the issues of disclosure, inconvenient access, difficulty in adherence, and high cost [19,37,38]. Given these disadvantages, daily PrEP was less endorsed by MSM in some studies. For example, the majority of MSM in Urumqi, China, preferred on-demand PrEP to daily PrEP [15], while an online survey in the United States showed that the number of MSM who were willing to use LAI-PrEP was three times that of the number willing to use daily oral PrEP [39]. However, participants in some other studies showed a higher willingness to use daily oral PrEP than the other two types due to its flexibility [40,41]. No consensus has been reached on which type of PrEP is more acceptable as the preferences are influenced by various factors, including demographic characteristics (e.g., income), sexual behaviors, and understanding of PrEP [42]. Therefore, to better understand which type of PrEP is more acceptable, it is essential to deliver accurate information of PrEP and provide individualized advice through education programs.

Participants in the present study self-reported high intention to adhere to PrEP if they started taking PrEP, especially on-demand PrEP. Identified factors associated with high adherence to PrEP in the literature include HIV risk perception and HIV literacy [43,44]. A published study developed a deterministic mathematical model to assess the epidemiological impact of PrEP and other biomedical interventions in China in the next 20 years. The mathematical model study showed that, even with low adherence (30%), a PrEP strategy targeting high-risk MSM can still reduce 10% of HIV infections within 20 years [45], suggesting that PrEP is still a promising strategy for controlling the HIV epidemic among MSM and should be recommended in conjunction with adherence programs [46].

Our study found that risky sexual behaviors, including multiple sexual partners and inconsistent condom use, were associated with the willingness to use and intention to adhere to PrEP among MSM. Participants who had more non-regular partners and reported inconsistent condom use with regular partners were more likely to say that they would use PrEP and adhere to PrEP, which is consistent with previous studies [30,39]. Risky sexual behaviors were facilitating factors for PrEP usage; therefore, programs focusing on MSM with risky sexual behavior, as an entry point of PrEP implementation, would be more likely to succeed and would be more cost-effective [47].

PrEP usage and condom use presented a paradoxical relationship in the present study. Participants who used a condom inconsistently with sexual partners were more likely to use PrEP to decrease the chance of HIV infection, but PrEP usage further increased the chance of inconsistent condom use as a risk compensation to increase the chance of HIV infection [48]. Inconsistent condom use following PrEP usage will reduce the benefits of PrEP directly, by increasing the risk of HIV transmission, and indirectly, by increasing the risk of transmission of other STDs [49]. Therefore, PrEP should be regarded as a supplementary intervention measure rather than a substitute intervention of condom use among MSM.

We did not find significant associations between alcohol consumption and the willingness to use and intention to adhere to PrEP among MSM, which is consistent with some previous studies [50]. However, other studies have argued that substance use leads to risky sexual behavior, which in turn affects PrEP use [51,52]. Different types of substances (e.g., alcohol, drugs) might produce different effects on PrEP use [53], and further studies are suggested.

The present study is subject to several limitations. First, the present study assessed the willingness to use PrEP among MSM on the condition of free PrEP, so findings may not be generalizable to the “real” setting of accessing PrEP at a high cost. Findings based on this sample recruited in Chengdu might not be generalizable to MSM living in other parts of China and worldwide. Second, we did not measure the actual adherence to PrEP among MSM who were already on PrEP; rather, we only measured the intention to adhere to PrEP among MSM who were not on PrEP. Self-reported bias and social desirability bias might exist, and we should be cautious in the interpretation of the findings. Third, we did not explore in detail the reasons regarding why participants preferred a certain type of PrEP, and a qualitative analysis might therefore be helpful. Fourth, we measured some key variables with a single item, including risk perception and risk compensation. Further improvement on these measures is suggested.

Despite the above limitations, a strength of the present study is that it is one of the first studies to investigate the willingness to use three different types of PrEP and the intention to adhere to PrEP usage among Chinese MSM. These three types of PrEP vary in doses, methods (convenience, burdens, difficulties in adherence) to use, potential side effects, concerns on disclosure of same-sex behavior, and efficacy. Therefore, this detailed and differential study on the level of willingness could better inform service planning than some of the existing literature that did not separate different types of PrEP.

## 5. Conclusions

This is one of the very few studies investigating the willingness to use and intention to adhere to three types of PrEP among Chinese MSM. We found the overall willingness to use free PrEP to be high after a brief introduction to PrEP, but willingness varied across the different types of PrEP. Having non-regular partners and higher HIV literacy were facilitators of the willingness to use PrEP, while consistent condom use with regular partners was a barrier. Keeping good adherence to PrEP and reducing inconsistent condom use as a risk compensation to PrEP usage are also two key issues along with PrEP implementation among MSM. Programs aiming to promote PrEP uptake and improve adherence among MSM should pay attention to the important roles of (1) delivering accurate information about HIV and PrEP to help MSM have higher HIV literacy and a better understanding of PrEP; (2) developing risk assessment tools to improve the accuracy of risk perception among MSM, especially for those who have risky sexual behaviors; (3) reducing forgetfulness and reminding medication by mobile-based app or other electronic devices; and (4) making more efforts to reduce social and self-stigmatization toward MSM.

## Figures and Tables

**Table 1:** Characteristics of participants (*N* = 524).

Measurement	Variables	Response Categories	Frequency	Percentage (%)
Background characteristics	Age (years old)	18–25	266	50.9
		26–45	238	45.5
		>45	19	3.6
	Ethnicity	Han	506	96.6
		Others	18	3.4
	Education level	Below university	202	38.5
		University or above	322	61.5
	Marital status	Married	47	8.9
		Unmarried	477	91.0
	Personal monthly income (CNY)	≤1000	58	11.1
		1001–3000	107	20.4
		3001–6000	197	37.6
		>6000	162	30.9
	Job type	Full-time	338	64.5
		Part-time	22	4.2
		Student	133	25.4
		Unemployed/Retired	31	5.9
	Sexual orientation	Homosexual	404	77.1
		Heterosexual	3	0.6
		Bisexual	91	17.4
		Not sure	26	5.0
	History of STDs	No	485	92.6
		Yes	39	7.4
	Risk perception for HIV infection	Moderate/low/very low	432	82.4
		High/very high	92	17.6
	Risk perception for STDs	Moderate/low/very low	450	85.9
		High/very high	74	14.1
	Drinking status	Non-drinking	278	53.1
		Drinking	246	46.9
	Binge drinking	No	427	81.5
		Yes	97	18.5
Risky sexual behaviors	Age of first intercourse (years old)	<18	92	17.6
		≥18	430	82.4
	Number of regular sexual partners in the past 6 months	0	126	24.0
		1	271	51.7
		>1	127	24.2
	Number of non-regular sexual partners in the past 6 months	0	168	32.1
		1–2	210	40.1
		3–5	105	20.0
		6–10	34	6.5
		>10	7	1.3
	Consistent condom use in the past month with all regular partners (*N* = 295)	No	112	38.0
		Yes	183	62.0
	Consistent condom use in the past month with all non-regular partners (*N* = 195)	No	48	24.6
		Yes	147	75.4
	Insertive and receptive anal sex with partners in the past month	No partner	107	20.4
		Insertive sex only	210	40.1
		Receptive sex only	151	28.8
		Both insertive and receptive sex	56	10.7
			**Mean**	**(SD)**
HIV literacy ^	Overall scale (range: 0–12)		9.4	(1.89)
	HIV prevention (range: 0–3)		2.6	(0.60)
	HIV diagnosis (range: 0–3)		2.6	(0.75)
	HIV treatment (range: 0–6)		4.2	(1.22)

STDs: sexually transmitted diseases, ^ the Cronbach’s alpha was 0.62 in the present sample.

**Table 2:** Descriptive statistics of pre-exposure prophylaxis (PrEP)-related variables (*N* = 524).

Variables	Response Categories	Frequency	Percentage (%)
Heard of PrEP	No	248	47.3
	Yes (any type)	276	52.7
	Daily oral PrEP	177	33.8
	On-demand PrEP	161	30.7
	LAI-PrEP	37	7.1
Overall willingness to use PrEP	No	79	15.1
	Yes	445	84.9
Willingness to use daily oral PrEP	No	209	39.9
	Yes	315	60.1
Willingness to use on-demand PrEP	No	109	20.8
	Yes	415	79.2
Willingness to use LAI-PrEP	No	195	37.2
	Yes	329	62.8
Intention to adhere to daily oral PrEP	Low intention	156	29.8
	High intention	368	70.2
Intention to adhere to on-demand PrEP	Low intention	79	15.1
	High intention	445	84.9
Intention to adhere to LAI-PrEP every 8 weeks	Low intention	151	28.8
	High intention	373	71.2
Risk compensation (condomless use)	Yes	226	43.1
	No	298	56.9
Acceptable price for PrEP (CNY)	0	58	11.1
	≤600 per month	315	60.1
	>600 per month	151	28.8

**Table 3:** Association between variables and the willingness to use any PrEP.

Measurement	Variables	Response Categories	Willingness to Use Any PrEP ORu (95% CI)	Willingness to Use Any PrEP AOR (95% CI)
Background characteristics	Age	≤25	1.00	
		>25	0.61 (0.38, 1.00) *	
	Ethnicity	Han	1.00	
		Others	1.44 (0.32, 6.37)	
	Education level	Below university	1.00	
		University or above	0.86 (0.52, 1.41)	
	Marital status	Married	1.00	
		Others	1.24 (0.51, 3.01)	
	Personal monthly income (CNY)	≤3000	1.00	
		>3000	0.70 (0.41, 1.21)	
	Job type	Full-time	1.00	
		Others	1.23 (0.73, 2.04)	
	Sexual orientation	Homosexual	1.00	
		Others	0.55 (0.32, 0.92) *	
	History of STDs	No	1.00	
		Yes	2.23 (0.67, 7.43)	
	HIV risk perception	Moderate/low/very low	1.00	
		High/very high	1.22 (0.63, 2.37)	
	STDs risk perception	Moderate/low/very low	1.00	
		High/very high	1.33 (0.63, 2.80)	
	Drinking status	Non-drinking	1.00	
		Drinking	1.64 (1.00, 2.69) *	1.56 (0.95, 2.56) †
	Binge drinking	No	1.00	
		Yes	1.32 (0.68, 2.55)	
Risky sexual behaviors	Age of first intercourse	<18	1.00	
		≥18	0.41 (0.18, 0.92) *	0.47 (0.21, 1.07) †
	Number of regular sexual partners in the past 6 months	0	1.00	
		1	0.87 (0.49, 1.55)	
		>1	1.43 (0.68, 3.00)	
	Number of non-regular sexual partners in the past 6 months	0	1.00	
		1–2	2.24 (1.29, 3.88) **	2.55 (1.45, 4.48) **
		3–5	2.58 (1.26, 5.31) **	2.85 (1.37, 5.93) **
		>5	2.80 (0.94, 8.33) †	3.36 (1.10, 10.26) *
	Consistent condom use in the past month with all regular partner(s)	No	1.00	
		Yes	0.47 (0.24, 0.95) *	0.47 (0.23, 0.95) *
	Consistent condom use in the past month with all non-regular partner(s)	No	1.00	
		Yes	0.74 (0.24, 2.35)	
	Insertive and receptive anal sex with partners in the past month	Insertive sex only	1.00	
		Receptive sex only	1.52 (0.82, 2.84)	
		Both insertive and receptive sex	0.89 (0.41, 1.93)	
HIV literacy	Overall		1.11 (0.99, 1.25) †	1.09 (0.97, 1.22)
	HIV prevention		1.51 (1.04, 2.18) *	1.49 (1.03, 2.16) *
	HIV recognition		1.06 (0.78, 1.45)	
	HIV treatment		1.14 (0.95, 1.38)	
Heard of PrEP	No		1.00	
	Yes		1.78 (1.10, 2.90) *	1.63 (0.99, 2.68) †
Risk compensation	Yes		1.00	
	No		1.00 (0.61, 1.61)	

ORu: univariate odds ratios, AOR: adjusted odds ratios, † *p* < 0.1, * *p* < 0.05, ** *p* < 0.01.

**Table 4:** Association between variables and adherence to PrEP.

Measurement	Variables	Response Categories	High Adherence to Daily Oral PrE PORu (95% CI)	High Adherence to on-Demand PrEP ORu (95% CI)	High Adherence to LAI-PrEP ORu (95% CI)
Background characteristics	Age	≤25	1.0	1.0	1.0
		>25	0.59 (0.41, 0.87) **	0.51 (0.31, 0.83) **	0.67 (0.46, 0.98) *
	Ethnicity	Han	1.0	1.0	1.0
		Others	0.84 (0.31, 2.29)	1.44 (0.32, 6.37)	1.43 (0.46, 4.43)
	Education level	Below university	1.0	1.0	1.0
		University or above	0.92 (0.63, 1.36)	1.50 (0.92, 2.42)	1.12 (0.76, 1.64)
	Marital status	Married	1.0	1.0	1.0
		Others	1.26 (0.64, 2.50)	0.63 (0.30, 1.32)	0.95 (0.49, 1.83)
	Personal monthly income (CNY)	≤3000	1.0	1.0	1.0
		>3000	0.61 (0.40, 0.93) *	0.65 (0.37, 1.13)	0.68 (0.45, 1.04) †
	Job type	Full-time	1.0	1.0	1.0
		Others	1.15 (0.77, 1.70)	1.41 (0.84, 2.37)	1.31 (0.88, 1.97)
	Sexual orientation	Homosexual	1.0	1.0	1.0
		Others	0.77 (0.50, 1.18)	0.63 (0.37, 1.07) †	0.80 (0.51, 1.24)
	History of STDs	No	1.0	1.0	1.0
		Yes	0.74 (0.37, 1.46)	3.49 (0.82, 14.79) †	1.19 (0.56, 2.50)
	HIV risk perception	Moderate/low/very low	1.0	1.0	1.0
		High/very high	1.43 (0.85, 2.41)	0.81 (0.44, 1.48)	0.97 (0.59, 1.59)
	STDs risk perception	Moderate/low/very low	1.0	1.0	1.0
		High/very high	1.08 (0.63, 1.86)	1.16 (0.57, 2.37)	1.03 (0.60, 1.77)
	Drinking status	Non-drinking	1.0	1.0	1.0
		Drinking	0.90 (0.62, 1.32)	1.07 (0.66, 1.73)	0.89 (0.61, 1.30)
	Binge drinking	No	1.0	1.0	1.0
		Yes	1.28 (0.77, 2.10)	0.96 (0.52, 1.78)	1.13 (0.69, 1.86)
Risky sexual behaviors	Age of first intercourse	<18	1.0	1.0	1.0
		≥18	0.56 (0.33, 0.97) *	0.56 (0.27, 1.16)	0.59 (0.34, 1.01) †
	Number of regular sexual partners in the past 6 months	0	1.0	1.0	1.0
		1	1.35 (0.86, 2.13)	0.98 (0.56, 1.72)	1.15 (0.73, 1.82)
		>1	1.21 (0.71, 2.06)	1.92 (0.90, 4.09) †	1.56 (0.90, 2.71)
	Number of non-regular sexual partners in the past 6 months	0	1.0	1.0	1.0
		1–2	1.01 (0.64, 1.57)	1.67 (0.95, 2.92) †	1.52 (0.97, 2.38) †
		3–5	0.98 (0.58, 1.68)	1.31 (0.68, 2.52)	1.23 (0.72, 2.08)
		>5	0.71 (0.35, 1.46)	1.69 (0.62, 4.66)	1.59 (0.73, 3.48)
	Consistent condom use in the past month with all regular partner(s)	No	1.0	1.0	1.0
		Yes	0.68 (0.40, 1.17)	0.49 (0.24, 0.99) *	1.05 (0.62, 1.77)
	Consistent condom use in the past month with all non-regular partner(s)	No	1.0	1.0	1.0
		Yes	0.92 (0.44, 1.95)	0.78 (0.28, 2.23)	1.10 (0.53, 2.31)
	Insertive and receptive anal sex with partners in the past month	Insertive sex only	1.0	1.0	1.0
		Receptive sex only	1.63 (1.00, 2.66) *	1.43 (0.77, 2.64)	1.43 (0.89, 2.30)
		Both insertive and receptive sex	0.85 (0.46, 1.60)	0.71 (0.34, 1.48)	1.48 (0.75, 2.94)
HIV literacy	Overall		1.04 (0.95, 1.15)	1.15 (1.02, 1.29) *	1.07 (0.97, 1.18)
	HIV prevention		1.20 (0.89, 1.64)	1.35 (0.93, 1.97)	1.27 (0.93, 1.73)
	HIV recognition		0.97 (0.76, 1.25)	1.25 (0.93, 1.67)	0.94 (0.73, 1.22)
	HIV treatment		1.07 (0.92, 1.25)	1.20 (1.00, 1.45) *	1.13 (0.97, 1.32)
Heard of PrEP	No		1.0	1.0	1.0
	Yes		1.68 (1.15, 2.45) **	1.58 (0.97, 2.56) †	1.23 (0.84, 1.80)
Risk compensation	Yes		1.0	1.0	1.0
	No		1.38 (0.95, 2.01) †	1.06 (0.65, 1.71)	1.40 (0.96, 2.04) †

ORu: Univariate odds ratios, † *p* < 0.1, * *p* < 0.05, ** *p* < 0.01.

**Table 5:** Multivariate analysis of association between variables and adherence to PrEP.

Variables	Response Categories	High Adherence to Daily Oral PrEP AOR (95% CI)	High Adherence to on-Demand PrEP AOR (95% CI)	High Adherence to LAI-PrEP AOR (95% CI)
Age of first intercourse	<18	1.0	-	1.0
	≥18	0.63 (0.36, 1.10)	-	0.63 (0.36, 1.09) †
Consistent condom use in the past month with all regular partner(s)	No	-	1.0	-
	Yes	-	0.48 (0.24, 0.98) *	-
Insertive and receptive anal sex with partners in the past month	Insertive sex only	1.0	-	-
	Receptive sex only	1.47 (0.89, 2.42)	-	-
	Both insertive and receptive sex	0.77 (0.41, 1.47)	-	-
HIV literacy	Overall	-	1.13 (1.00, 1.26) *	-
	HIV treatment	-	1.18 (0.98, 1.42) †	-
Heard of PrEP	No	1.0	-	-
	Yes	1.67 (1.14, 2.44) **	1.54 (0.95, 2.51) †	-
Risk compensation	Yes	1.0	-	1.0
	No	1.43 (0.98, 2.09) †	-	1.43 (0.98, 2.10) †

AOR: Adjusted odds ratios, † *p* < 0.1, * *p* < 0.05, ** *p* < 0.01.
